# A serious game to explore human foraging in a 3D environment

**DOI:** 10.1371/journal.pone.0219827

**Published:** 2019-07-25

**Authors:** Valter Prpic, Isabelle Kniestedt, Elizabeth Camilleri, Marcello Gómez Maureira, Árni Kristjánsson, Ian M. Thornton

**Affiliations:** 1 Institute for Psychological Science, Faculty of Health and Life Sciences, De Montfort University, Leicester, United Kingdom; 2 Institute of Digital Games, University of Malta, Msida, MSD, Malta; 3 Faculty of Psychology, School of Health Sciences, University of Iceland, Oddi v. Sturlugötu, Reykjavik, Iceland; 4 School of Psychology, National Research University, Higher School of Economics, Moscow, Russian Federation; 5 Department of Cognitive Science, Faculty of Media and Knowledge Sciences, University of Malta, Msida, MSD, Malta; University of Plymouth, UNITED KINGDOM

## Abstract

Traditional search tasks have taught us much about vision and attention. Recently, several groups have begun to use multiple-target search to explore more complex and temporally extended “foraging” behaviour. Many of these new foraging tasks, however, maintain the simplified 2D displays and response demands associated with traditional, single-target visual search. In this respect, they may fail to capture important aspects of real-world search or foraging behaviour. In the current paper, we present a serious game for mobile platforms, developed in Unity3D, in which human participants play the role of an animal foraging for food in a simulated 3D environment. Game settings can be adjusted, so that, for example, custom target and distractor items can be uploaded, and task parameters, such as the number of target categories or target/distractor ratio are all easy to modify. We are also making the Unity3D project available, so that further modifications can also be made. We demonstrate how the app can be used to address specific research questions by conducting two human foraging experiments. Our results indicate that in this 3D environment, a standard feature/conjunction manipulation does not lead to a reduction in foraging runs, as it is known to do in simple, 2D foraging tasks.

## Introduction

When humans or animals explore an environment, their behavior is guided by a variety of factors. These may include internal goals (e.g., hunger, arousal), internal constraints (e.g. memory capacity, available attentional resources), or external aspects of the scene, such as overall lighting conditions or salient visual features. Traditionally, two largely separate disciplines have been used to explore these factors. In humans, “visual search” studies typically use simplified displays in which a single target item is presented in the context of a varying number of distractor items. [1–4]. In other species, more complex “foraging” behavior has been studied—often involving rich environments, extended search episodes and multiple target items—using a variety of paradigms, both in the laboratory and in the wild [5–9].

The purpose of the current paper is to describe our initial findings with a new research tool that incorporates aspects of both traditional visual search and animal foraging paradigms. Specifically, we have developed a serious game for mobile platforms, in which human participants play the role of an animal foraging for food in a simulated 3D environment. As described in more detail below, we are making the game freely available and are also providing access to the Unity3D source project, so that other groups can adapt the simulated environment and task to match their research interests.

**Fig 1:**
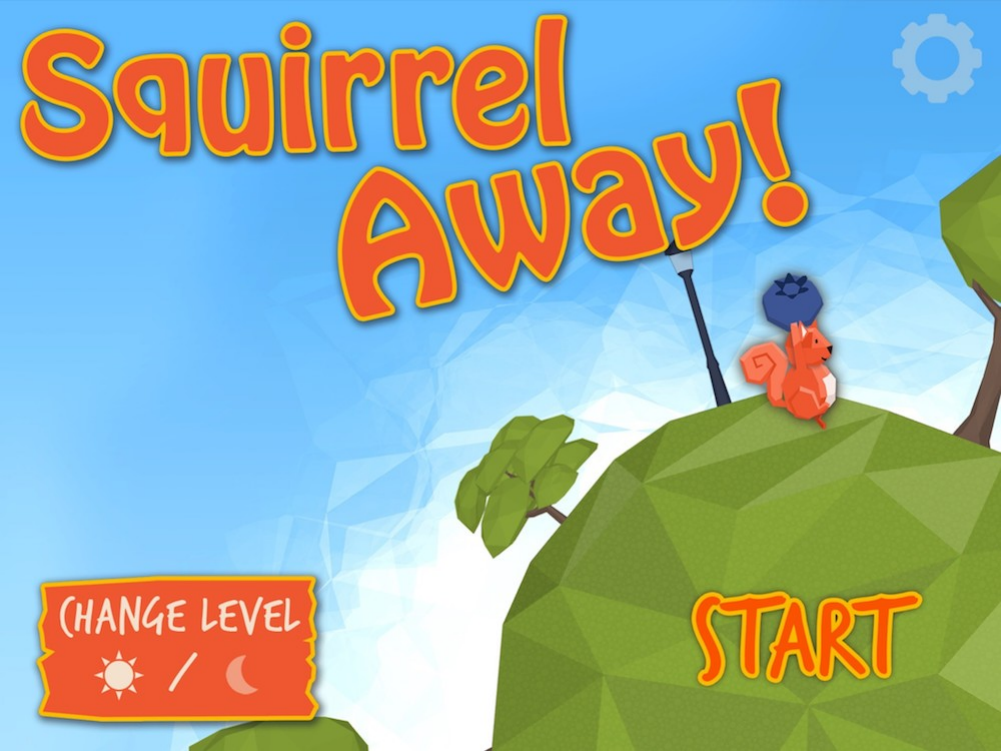
Title screen from the serious game “Squirrel Away”. A screen shot from the initial game title scene.

**Fig 2:**
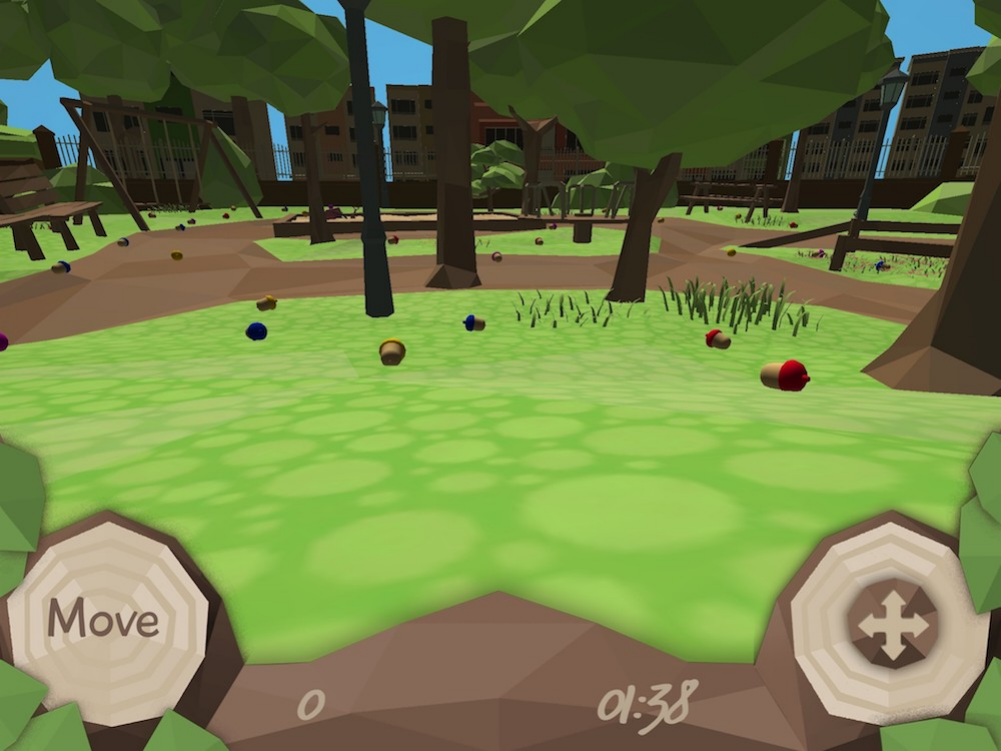
Screen shot from in-game play.

**Fig 3:**
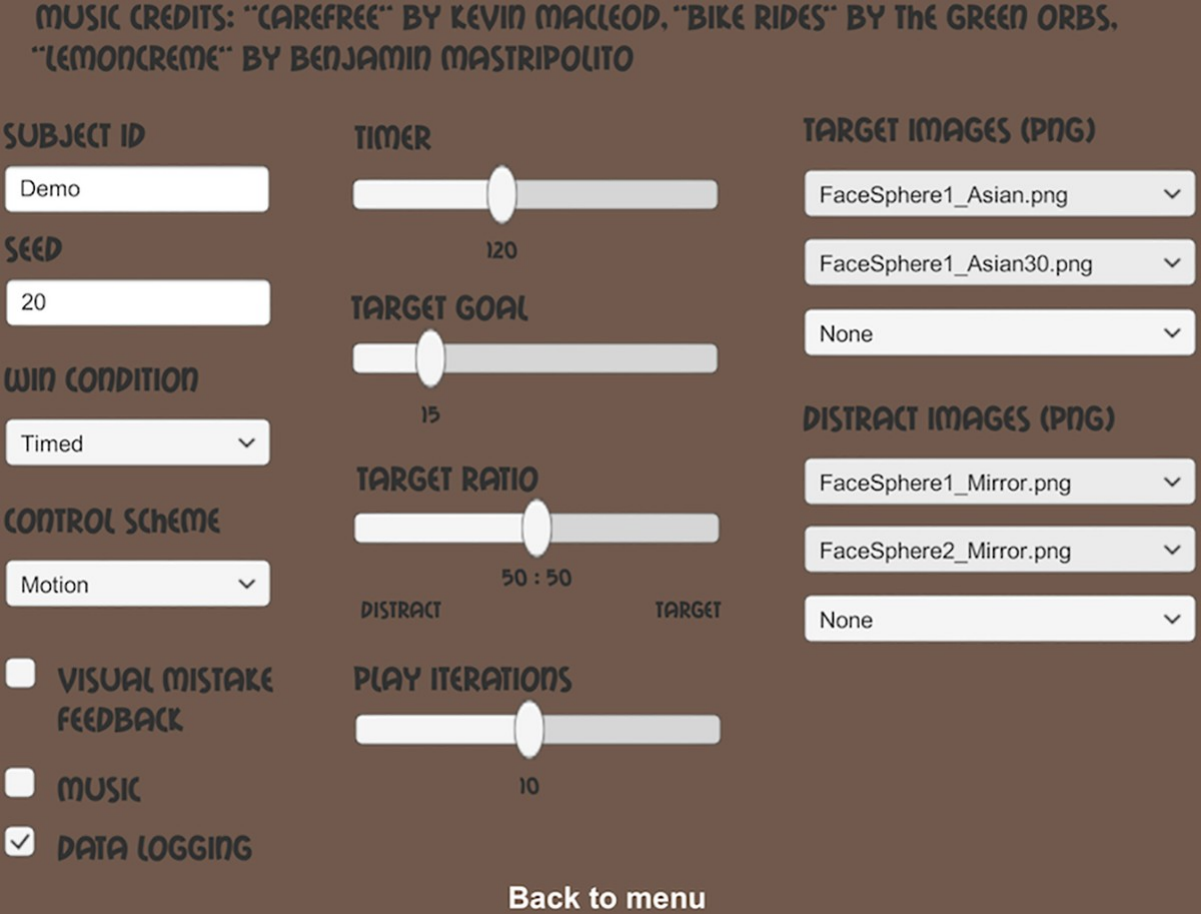
App settings menu. From this screen, the researcher can control many aspects of the game play and experimental settings. Details are provided in the text, but note that the “Target” and “Distractor” images interfaces to the right of the screen make it possible to load fully customised stimuli into the app. This feature makes it easy to vary both search context and task difficulty. Other parameters that can be varied include the length of a game session, either in terms of number of collected items or time, the ratio of target to distractor items, and whether data is being logged.

Fig 1 shows the title screen of the game and Fig 2 a snapshot from a typical game scene, while in S1 and S2 Movies it is possible to see the game in action in the feature and conjunction conditions, respectively. From within the app, the game environment can be easily adapted by the researcher. For example, the identity of target items can be fully customized, and task parameters, such as target/distractor ratio, number of trials and trial duration are all easily modified (Fig 3). Data relating to a wide range of foraging behavior is directly recorded on the device and can easily be accessed in standard text format.

The screen shot in Fig 2 shows the first-person perspective of the player, with target items distributed relatively sparsely ahead. The lower portion of the screen also shows the control and information display bar. Here, with the traditional user interface, there are two control buttons. By pressing and holding the “Move” button, the player moves through the scene, collecting items by colliding with them. The right button is a virtual joystick that can be used to control heading. When the optional motion-based user interface is selected, the joystick is absent and heading is controlled using the on-board gyroscope as the user makes full-body movements of the device in space. A countdown timer and a running counter of the number of collected targets are also displayed for information.

In the following sections, we provide background information on serious games and the specific research questions that prompted us to develop the current tool. We then provide a detailed description of the app and present data from two initial experiments where we used it to ask a specific question about human foraging. We conclude with a discussion of our findings and suggestions for future studies and possible modifications of the app.

### Serious games

The term “serious games” refers to full-fledged games with a purpose other than entertainment [10]. This sets them apart from regular games, whose primary purpose is entertainment, and from gamification, which refers to the use of game elements in non-gaming contexts [11]. Serious games leverage the positive effects of videogames, such as entertainment value or high levels of engagement and/or immersion, in order to achieve a particular objective. Most commonly, this objective relates to learning or training. Common applications of serious games can be seen in domains of education [12], marketing [13], health [14], and the military [15].

Another increasingly common use of serious games is within various academic disciplines, most notably within psychology [16–18]. Here, games—particularly when used in tandem with mobile devices [19]—are thought to have great potential as research tools [20–22]. One primary benefit of serious games is that they can be highly interactive. They can be used to place the participant in a virtual environment in which certain behaviour may be elicited through careful design of game mechanics–design aspects that invite, encourage, or discourage certain behaviour to achieve the game’s goals. Utilizing aspects of entertainment games, they are furthermore seen to be more engaging than standard experiment tasks. They also allow for real-time data collection of in-game behaviour [23]. Finally, they can be used to simulate situations that are physically difficult or even impossible to study, and to provide safe, virtual scenarios that could be deemed unethical when studied in reality.

As we discuss in more detail below, one possible consequence of using serious games for research, is that the display and interaction dynamics often become very complex compared to traditional experimental tasks. For example, even when all underlying mechanics are known to the researchers, the way that elements in a game interact, and are interacted with by the player, may still give rise to unpredictable outcomes. This can make it potentially difficult to isolate the factors directly influencing behavior.

In some contexts, it may be preferable to use “incremental” gamification, where individual game elements–such as leaderboards, realistic interaction or more engaging graphics–are added in stages to a standard experimental task. Of course, as more game elements are added, the line between a fully-fledged game and a gamified task soon becomes blurry. The goal of the current paper is not to argue for one approach over the other, as this will clearly depend on specific research questions and contexts. Here, we opted for the design of a serious game that makes it possible to initially observe and manipulate foraging behaviour in a complex environment, acknowledging the fact that additional steps may be required in order to isolate causal factors.

### Background

In our previous work [24], we have already explored the use of simple, 2D games as a way to move beyond traditional studies of visual search. Classic visual search studies have taught us a great deal about vision and attention [1,3,4,25,26]. However, these tasks typically involve only a single target, and search ends once the target has been found or observers decide that no target is present. Search behavior in the real-world is often more complex than this, for example, involving multiple target episodes with less clearly defined end states. Traditional studies, with their limited selection scenarios, may therefore offer only a limited picture of attentional selection in the wider environment.

To address these concerns, a number of labs have sought inspiration from the animal literature mentioned above and have begun to investigate how humans “forage” for multiple targets [24,27–34]. Overall, the findings seem consistent with the idea that there is a common evolutionary thread that links the foraging behavior of humans and animals in complex scenarios. For example, a number of key findings from the animal literature have been found to have parallels in human behavior, including search in extended “runs” [24], Lèvy flights [31], the predictions of Marginal Value Theorem [34] and “area-restricted search” [35].

Our own interest in this area was stimulated by the classic laboratory study of Marian Dawkins [5]. She demonstrated that when feeding, chicks often selected the same type of food item in long “runs”, rather than selecting at random from two available categories. A “run” simply refers to a sequence of selections from the same target category. In general, when there are many short runs, this suggests that an animal is selecting at random. Fewer, longer runs, suggest that feeding is being guided in some way. Nikolaas Tinbergen [36,37] had previously noted the tendency towards long-run behavior in wild-feeding birds, leading him to postulate that their behavior was being guided by some form of internal “search-image”, see [38] for discussion. Later work suggested that reliance on such templates might increase when preys were cryptic or attentional demands increased for other reasons, such as fear of predation [39–42].

**Fig 4:**
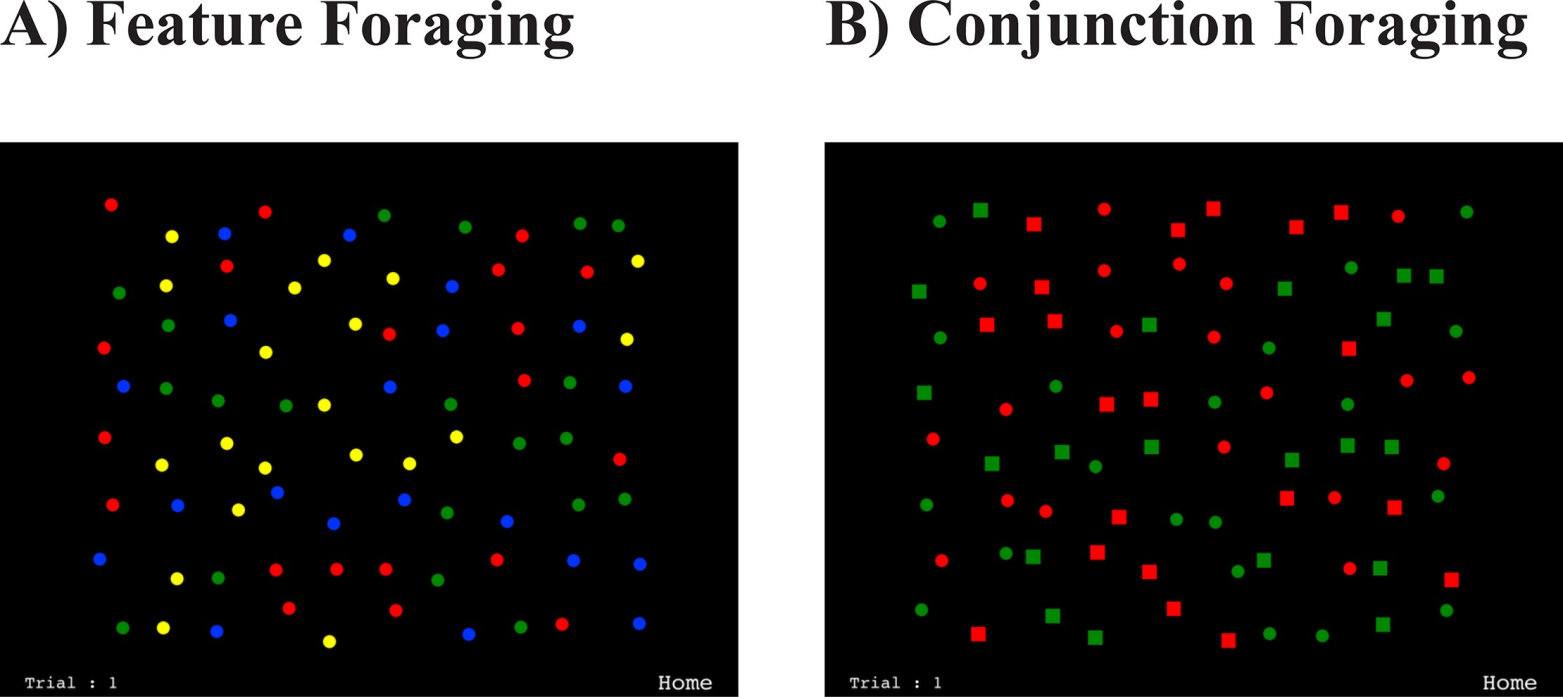
Stimuli used in the previous 2D foraging iPad tasks. Examples of the feature A) and conjunction B) foraging, respectively. In previous studies participants were asked to cancel items from two target categories (e.g., red & green dots in A, red circles, green squares in B) as quickly as possible by tapping items with their fingers.

The 2D foraging game we created for humans conceptually replicated the study of Dawkins [5]. As can be seen in Fig 4, we did this by presenting multiple target stimuli on an iPad [24]. Observers were required to tap on target dots from two categories (e.g., red and green dots) while ignoring stimuli from distractor categories (e.g. blue & yellow dots). Tapping on a target made it disappear, and the task was to forage until all targets had been cancelled. Participants found the task engaging and intuitive, and typically completed a trial (i.e. cancelling all 40 targets) in well under 20 seconds.

**Fig 5:**
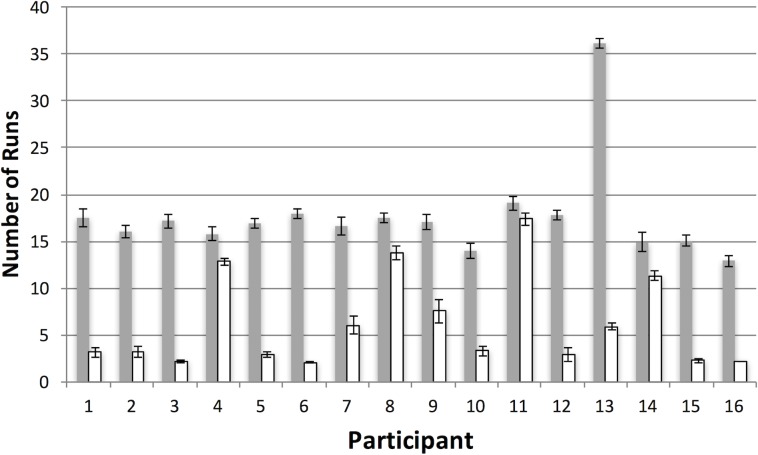
Average number of runs for each participant in one of our previous studies [24]. The data show that feature foraging typically involves frequent switches between the two target categories, giving rise to many runs (grey bars), whereas for most participants, conjunction foraging involves far fewer runs (white bars). This difference between feature and conjunction conditions has been replicated a number of times, and is thought to reflect changes in foraging strategy caused by an increase in attentional load. Error bars represent one standard error of the mean.

During feature-based foraging (Fig 4A), where the two target categories differed from the two distractor categories only in terms of a single feature (i.e. colour), participants switched randomly between target types. This gave rise to many short runs, suggesting random selection from the two target categories (black bars, Fig 5). The pattern was very different during conjunction-based foraging (Fig 4B) where the attentional load was increased as targets differed from distractors in terms of two features [1]. For example, the targets could be red squares and green dots, while the distractors were green squares and red dots, or vice versa. In this case, the majority of participants foraged using far fewer runs, repeatedly selecting for the same target category, rather than switching between categories (white bars, Fig 5). This indicates that they had trouble using more than one conjunction template to guide their selection. Thus, with a simple, game-like task, we were able to provide the first demonstration of extensive run-like behavior in humans, suggesting that human foraging may be constrained by attention in the same way as it is with animals [40,41].

### Motivation for current work

While the simple feature-conjunction manipulation we used in our 2D game had quite a dramatic effect on foraging behavior, the nature of the task itself clearly differs in many respects from foraging behavior that might take place in the wild. The general motivation for the current work was to create an experimental framework where we could begin to explore foraging in richer environments. In our 2D game, participants had a global view of all targets and distractors at all times and made selections approximately twice a second. As described in the next section, our 3D game aimed to more closely simulate the visual experience of a foraging animal by presenting a complex environment within which food items were quite sparsely distributed. In this environment, only a subset of items would ever be visible from a given vantage point and thus locating targets required relatively slow, active navigation through the scene, keeping track of the spatial layout and the current position while planning where to move next.

Foraging performance in such an environment may clearly be affected by many additional factors that are absent from simple 2D tasks. Active, spatial exploration of simulated environments is known to make demands on attention and working memory for example (see [36,37] for review), and may also be subject to consistent individual differences, e.g., [43]. The approach we have adopted in our initial experiments is to keep such factors constant across the main experimental manipulation of interest. Rather than confounding variables, they thus become contextual dimensions that have the potential to reveal useful behavioral findings. Our research question was specifically whether the conjunction manipulation used in our previous 2D studies would continue to strongly modulate performance during active exploration of a simulated 3D scene.

As we return to later, such a design necessarily means that, regardless of the outcome, subsequent studies–either in complex 3D or simplified 2D tasks–will be required to isolate which dimensions exert influence, and to more fully understand the nature of that influence. Our hope is that the serious game we describe next will provide a starting point where behavior can be observed (and measured) in a complex simulation, giving rise to initial insights that allow important factors to be isolated and further explored.

### App details

The serious game that was developed is a single-player tablet game for iOS called “Squirrel Away”, using the freely available game engine Unity3D. Based on the 2D task described earlier, we took the same “foraging” concept and applied it to a 3-dimensional, virtual environment. Since navigation of a 3D environment tends to be challenging for people who are inexperienced with videogames or other 3D software, making the game accessible was a key concern during the design. The way we approached this was through the use of simple to understand, straight-forward game mechanics and flexible control options.

In the game, the player takes on the role of a squirrel gathering food for its family. The player controls the squirrel from a first-person perspective and explores a park where ‘target’ and ‘distractor’ objects are spread across the environment. The default mode for the game has players on a two-minute timer in which they try to gather as many of the target objects as possible. Objects are collected upon collision, meaning players simply run into the objects to collect them. Collecting target objects grants points, while distractor objects temporarily restrain the player and negatively impact the score by resetting it to zero.

The game utilizes simple mechanics of points and time-constraints that are reminiscent of older-style ‘arcade’ videogames. In order to emphasize the exploratory aspects of the game, we created an interesting game-play experience by giving players the perspective of a small animal exploring a large environment. This was done through the scale and design of the virtual environment, the positioning of the camera and its field of view, the player’s movement speed, and the use of sound effects. The park environment simulated a 20 x 20 meter space, and speed of movement was constrained to be no higher than 0.7 meters/second.

We developed two control types for this game. The first involved an on-screen virtual joystick on the bottom right of the screen. This mimics a standard video game controller and control schemes found in commercial tablet-based 3D games. The second control type was motion-based, and involved the use of the tablet’s gyroscope to orient the first-person camera to where the player points the device. In this sense, it works as if one would take a picture using the tablet's camera, but instead of looking at real-life surroundings, the camera looks into the virtual environment instead. In practice, this has the player physically turning full circle to turn around in the game, as well as point the tablet up and down to look above or below in the game environment. We have found that participants find this method of control to be more intuitive, as it mimics the use of the camera that they are familiar with from their own devices. The only other control in the game is the ‘move’ button, which is located at the bottom left of the screen. By holding the button pressed the player will move forward in the direction the camera is facing. Releasing the button will make the player stop moving.

Besides choosing which controls to use, the game features a menu that allows the customization of several features. On the title page the researcher can change the game level from day to night; when playing at night the player’s vision will be limited due to low lighting levels. This also changes the music and sounds in the game. When going into the settings menu, the researcher can put in a subject ID for data logging purposes, as well as a seed number that controls the randomization of the collectible items (i.e. putting in the same number will result in the same random distribution between experiments). Other options include the length of a game session (with the default of two minutes), the amount of times the player will play a game session before returning to the main menu (i.e. game ‘loops’), the ratio of target to distractor items, and the target number of items to collect. The target amount is unused when the winning condition is set to ‘Timed’, in which case the player has until the timer runs out to collect as many items as they can. If the win condition is set to ‘Target’ the game ends once the player has collected the required number of items. When the win condition is set to ‘Both’, the game round will end either when the timer runs out or when the player has collected the target amount.

Notably, the researcher can load in images (in PNG format) for the target and distractor items, with a maximum of three images per category. These act as ‘billboards’ in the virtual environment, meaning that they are flat pictures that always face the camera. A trick from older videogames, this gives the effect of a 3-dimensional object within the virtual space, without requiring the design and importing of complex assets. Finally, the researcher can turn the music, as well as the data logging capabilities, on or off.

When data logging is turned on, the game will log the key events and player movements to a series of four .csv text files directly on the iPad. The files can be accessed and downloaded for analysis via iTunes File Sharing. Each filename includes a common identifier consisting of the date and time at which the game level was entered, the participant ID, if one has been entered, and a file label, indicating the nature of the recorded data (e.g., 16-05-2019 07_33_11.356-ExamplePPT-objectPositions.csv).

The “Object Positions” file is created at the start of each trial and records the precise layout of target and distractor items within the scene for that trial. Seven variables are recorded, specifically: the date and time (dd-MM-yyyy HH:mm:ss.ms) at which the game level was entered; the time from start (seconds, milliseconds) at which the trial layout was created; the trial number; whether each item is a target or distractor; the unique item identifier; a cluster identifier, which can be used to group adjacent items; the precise X, Y, Z position in Unity units (i.e. simulated meters).

The “Path” file records the player position (logged at 10 Hz) throughout the entire trial, so that this can be recreated precisely if need. Seven variables are recorded, specifically: a UTC timestamp (dd-MM-yyyy HH:mm:ss.ms); an internal framecount since the start of a trial; the trial number; current position within the scene (X, Y, Z); the current camera rotation (X, Y, Z); number of targets collected so far; number of distractor collisions so far.

The “Collection Order” file records each collection event involving either target or distractor items. Seven variables are recorded, specifically: a UTC timestamp (dd-MM-yyyy HH:mm:ss.ms) of when the event took place; a simplified event time since start of the trial (seconds, milliseconds); the trial number; whether item was a target or distractor; the unique item identifier; a cluster identifier, which can be used to group adjacent items; the precise X, Y, Z position of the collected item in Unity units (i.e. simulated meters).

Finally, the “Synch” file records the user settings at the start of each session, the time and type of each event that took place during a trial and a summary of performance at the end of a trial, in terms of number of collected targets, number of distractor collisions and total duration (seconds, milliseconds).

Example data files for the current experiments are included in the supplementary material. These data files were created from the raw log files, but have been additionally processed to extract the dependent measures of interest. Examples of raw log files are provided on the associated OSF page. We also note that changing the content or resolution of logged information should be relatively easy by amending the DataLogger.cs script of the provided Unity project.

## Access to the game & performance notes

As already noted, Squirrel Away has been optimized for deployment as an iPad app. At the time of writing, we are making the app available to other researchers via Apple’s *ad hoc* distribution pipeline. To obtain a copy of the working game used for the current studies, simply send an email to the corresponding authors specifying the iPad model, Unique Device ID (UDID) and iOS version of the device you intend to use. These parameters are all displayed on the “Summary” tab of iTunes, once the device is connected to a computer. Alt-clicking the serial number will reveal the UDID.

Once we receive this information, we can build a version of the app for your device and provide a download link via email. Alternatively, the Unity3D project can be downloaded from the Open Science Framework (OSF) page associated with this project https://osf.io/ef3cn/. This will make it possible for other groups to directly deploy the app to their devices as well as to modify and/or re-target the app for use with other platforms. We will also publish updates about the status of app and future downloadable versions on this OSF page.

We chose to conduct our research with iPads as the display and response characteristics of these devices have been well-documented elsewhere [44–46], and we have previously used them to obtain reliable RT data using a number of tasks [24,45,47,48]. Both the Unity3D and Xcode development environments contain a number of tools for optimization and analysis, and we used these during development to verify that the display and timing performance were within acceptable limits. Specifically, that displays were updated consistently at the desired rate of 30 Hz—providing smooth, jitter-free animation–that requested data was accurately logged at 10 Hz and that collision events were timed with millisecond precision.

Squirrel Away is not particularly graphics or CPU intensive and Unity3D is often used to produce commercial games that make much higher demands. In our opinion, Unity3D has the potential to become a useful, general-purpose experimental creation avenue for the research community, although we are unware of published performance benchmarks aimed at this community and clearly each task/device deployment would need to be evaluated separately.

## Experimental studies

To illustrate how the game can be used as a research tool, we conducted two laboratory-based experiments in which we varied the attentional demands of individual target selection using the same feature/conjunction manipulation used in our original 2D study [24]. As already noted, this manipulation consistently led to a very clear change in search strategies, such that participants selected randomly from target categories during feature conditions, yielding short runs of the selection of the same target type, but used long, non-random runs during conjunction conditions. Our current question was whether a similar shift would occur with a task that more closely approximates a real-world environment.

Again, we should be clear that these initial experiments were not designed to provide direct quantitative comparisons with our previous work, nor to isolate which aspects of the 3D simulation might critically affect behavior. However, it is clearly an important initial question to ask whether the same attentional manipulation that has such a strong influence in the 2D case has any influence on search behavior when viewing conditions and time constraints more closely approximate real exploration in a 3D environment.

### General methods

As the two experiments conducted here only differed in two small details, we will note these immediately, and then report the common aspects of the methodology. In Experiment 1, participants were seated and used virtual joysticks to orient themselves in the virtual world. In Experiment 2, they were standing, and used full-body movements to change their viewpoint. This change was made to explore whether the method of interaction affected performance. The second difference concerned the target/distractor ratio. In Experiment 1 there was a 50/50 target/distractor ratio in both feature and conjunction conditions. In Experiment 2, we changed the ratio in the conjunction condition to 30/70 in order to further increase the level of difficulty.

#### Ethics statement

All aspects of the experimental procedures were reviewed and approved by the relevant Faculty Research Ethics Committee at the University of Malta and thus conformed to the ethical guidelines set out by the Declaration of Helsinki for testing human participants. All participants gave written informed consent and were paid a previously agreed amount that reflected the standard payment in the department.

#### Participants

A total of 24 participants took part in the two experiments. In Experiment 1, 12 members of the University of Malta community took part in the study (8 females; 11 right handed; M = 23.7 years, SD = 4.8 years, range 19–33). In Experiment 2, a further 12 participants from the same population took part (8 females; 12 right handed; M = 24.3 years, SD = 7.2 years; range 18–44 years). All participants reported normal or corrected to normal vision and none took part in both studies.

#### Equipment

The app was deployed to an iPad 2, which was used for stimulus presentation and data collection. The iPad has a screen dimension of 20 x 15 cm and an effective resolution of 1024 x 768 pixels. An Xcode project was created directly from the Unity 3D game environment and this was compiled to produce the iPad version of the app. The iPad was in landscape mode and it was held with both hands by the participants. Details of the virtual viewing dimensions are given above. In Experiment 1, participants were seated in a sound-attenuated booth with dim illumination. In Experiment 2, they were standing in a screened off area of the lab, again with low background illumination.

#### Stimuli

**Fig 6:**
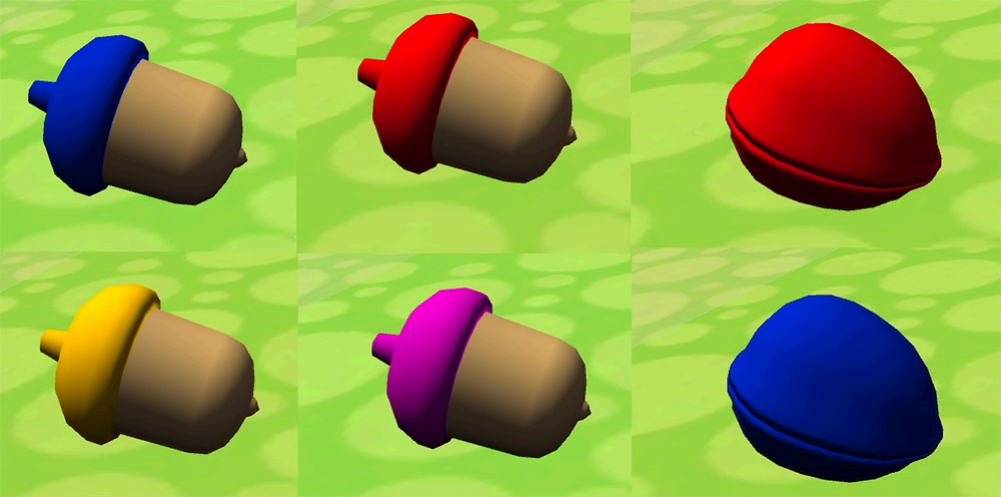
Target stimuli used in Experiments 1 & 2. During feature foraging, target items were red and blue acorns and distractors were yellow and purple acorns. For the conjunction condition, targets were red walnuts and blue acorns and distractors were blue walnuts and red acorns. These target categories conceptually replicated the conditions used in our previous 2D studies. Note that the app has been designed so that researchers can create their own target and distractor categories simply by loading new images from the Settings menu (see text for details).

In the current experiments Target and Distractor stimuli were food items in keeping with the overall theme of a foraging squirrel. As already noted, custom images of any kind can be loaded as part of the app setup menu. Here, in the feature condition, the targets were red and blue acorns and the distractors were yellow and purple acorns (see Fig 6). In the conjunction condition, the targets were red walnuts and blue acorns and the distractors were blue walnuts and red acorns.

**Fig 7:**
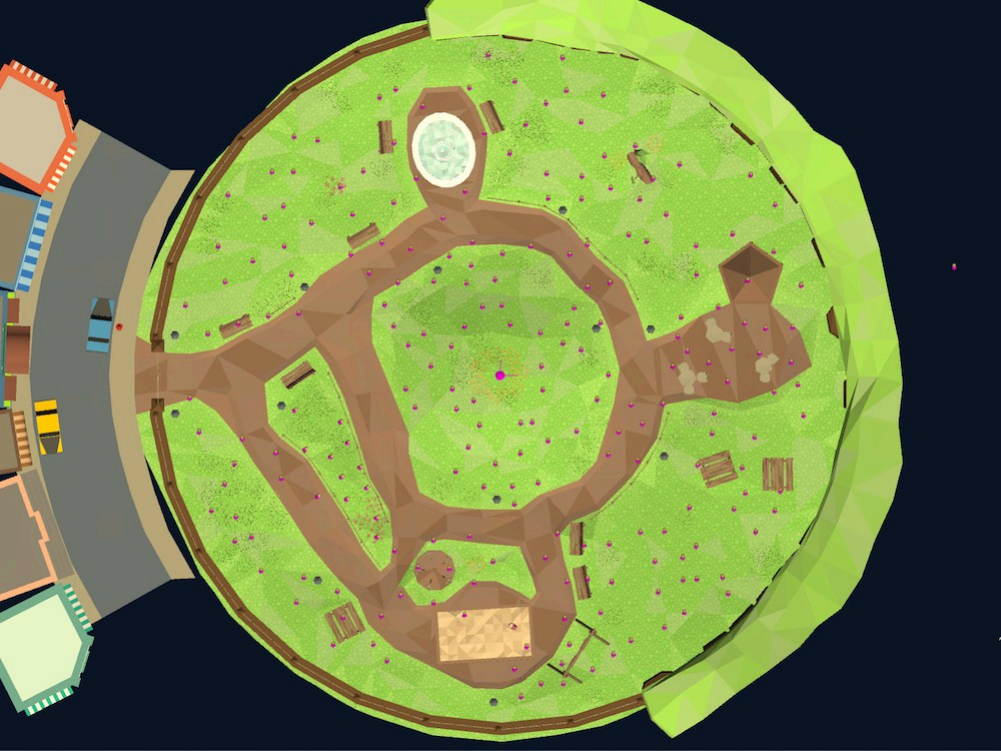
Plan view of the game layout. This view is never seen during game play, but is shown here to demonstrate the distribution of items throughout the park. Each dot indicates a possible item location, which become randomly populated at the start of each session. In this view, the sparse distribution resembles the layout of our 2D experiments (see Fig 4), although during 3D game play, only a sub-set is visible at one time (see Fig 2).

The stimuli were randomly distributed across the park scene using the grid structure shown in Fig 7. Note how this “plan view” of the 3D world is conceptually similar to the 2D distribution of target items in our original 2D task (Fig 4). In Experiment 1 there were approximately 200 items and the target/distractor ratio was 50/50, meaning there were equal numbers of each type of stimuli. In Experiment 2, the only change was that the target/distractor ratio was reduced to 30/70 in the conjunction condition.

#### Task

Participants were asked to play the mobile app game, "Squirrel Away". In this game, participants played the role of a squirrel searching for food in a 3D virtual environment. As described above, the game used a first-person perspective, thus participants experienced the viewpoint of the character as it moved through the park scene.

In Experiment 1, a virtual joystick and a ‘move’ button were displayed at the bottom of the screen to control movement. By keeping the left button pressed the character travelled forward in the 3D environment, by releasing the button the character stopped. The joystick on the right of the screen was used to change direction. In Experiment 2, the joystick was replaced by a motion interface, where direction was controlled by changing body position. Participants were standing with the device and changed the visible part of the scene as they turned to the left or right. Forward motion was controlled with the left button as before.

The goal of the task in both experiments was to collect 30 target items from either target category. The character had to be made to collide with stimuli in order to collect them. When a target was collected, audio feedback was provided and a target counter, displayed centrally at the bottom of the screen, updated the number of collected targets.

When a distractor was mistakenly collected, distinctive error audio feedback was provided and the target counter was set back to zero. As a consequence, the time required to complete the trial would greatly increase as participants would then have to search again for the required number of targets in the now reduced search space (items were not replaced after an error). In practice such errors were extremely rare (< 1% of total collection episodes) and our data analysis–for all dependent measures—only focused on the period in which the final 30 correct selection episodes in a given trial took place.

We chose this rather severe error penalty to approximate that used in our original 2D tasks, where selecting a distractor would typically terminate the entire trial. In the current scenario, as there were only limited number of trials, and participants were likely to be more “immersed” in the environment during the extended search period, it was felt that terminating the trial would be too disruptive. However, pilot testing suggested that resetting the collection history could serve as an equally strong deterrent.

We should note that in a very recent 2D study [49], we have shown that removing performance consequences when selecting a distractor, and simply counting the number of errors, has little or no impact on patterns of run behavior. Adopting a similar approach in further 3D studies with the current task would involve a very simple change to the provided Unity3D project.

#### Procedure

Both experiments consisted of three parts: a familiarization phase and two testing phases. We used a fixed order for the testing phases in which the feature condition always preceded the conjunction condition. We did this in order to more fully isolate the impact of increasing the attentional load in the conjunction condition. Pilot testing indicated that participants did switch randomly when targets were defined by a single feature. From previous visual search [1] and foraging studies [24], we had clear reason to assume that the conjunction condition would place higher perceptual demands on participants than the feature condition. We thus used the feature condition as our practiced baseline measure, and asked the specific question as to whether observed run behavior would change once more difficult target selection was required.

In the familiarization phase, participants were given the opportunity to experience the environment and the task controls. They were asked to collect 20 feature targets within a time limit of 2 minutes. Both a target counter and a 2-minute countdown were displayed at the bottom of the screen. Participants could take as many attempts as they needed and were allowed to proceed to the next phase of the experiment only when they were able to complete the task.

In the first testing phase, participants completed the feature foraging condition. On each trial they had to collect 30 targets without time limit. There were 5 trials to complete the whole phase. Although there was no explicit time limit, participants were instructed to complete the task as quickly as they could. A timer was displayed at the bottom of the screen and participants were encouraged to use 5 trials to improve their completion time. Accuracy was also stressed in the instructions and participants were aware that by collecting a distractor they would lose all the targets they had collected in the current trial.

In the second testing phase, participants completed the conjunction condition. As in the previous condition, they had to collect 30 targets to complete a trial. They were given one trial to practice with the new targets, and then completed 5 trials as before. Otherwise, task instructions were the same as in the previous phase.

Participants were allowed to take a break after every trial and every phase of the experiment. The total time required to complete the experiment varied, but was usually around 40 minutes.

## Results

The main dependent variable was the number of target “runs” produced on each trial [5,24]. As already mentioned, a run refers to a sequence of selections from the same target category. In an experiment where there are two target types, when the number of runs per trial approximates total-targets/2, this indicates that targets are being selected at random. Fewer runs per trial suggest category-based selection. In addition to the number of runs, we report two other measures that have been shown to vary as a function of the feature/conjunction manipulation: the average inter-target distance (ITD) and the average inter-target time (ITT) [50]. For all of our dependent variables our analysis was restricted to the final 30 correct collection episodes on each trial. Raw data on these collection episodes for all of the participants in Experiment 1 and 2 are available in S1 and S2 Files of the supplementary material, respectively.

**Fig 8:**
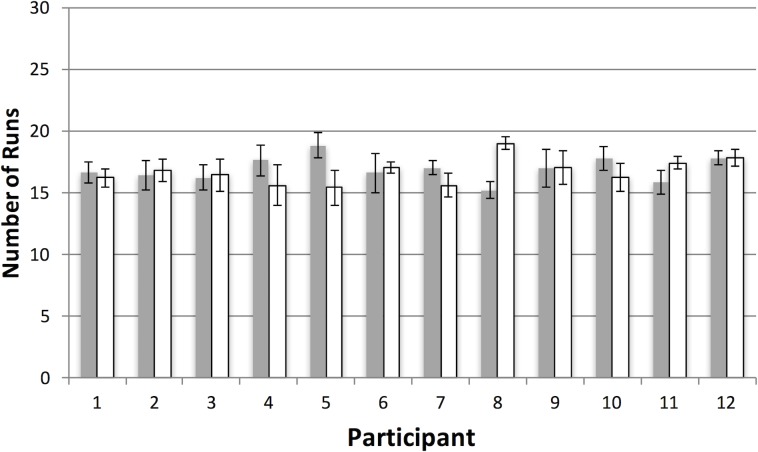
Results for number of runs in Experiment 1. Average number of runs per participant as a function of the feature (grey bars) versus conjunction (white bars) manipulation. In contrast to our previous 2D studies, there was no difference in proportion of runs in the two conditions. As there were 30 possible targets, an average number of runs of approximately 15 suggests that participants were switching between categories at random (see text for details). Error bars represent one standard error of the mean.

In general, participants appeared to explore widely in the virtual space. S3 Movie shows the spatial distribution of each collection episode for every trial in both conditions of Experiment 1. Fig 8 shows how these collection episodes were organized in terms of runs. There are two points to note. First, average runs, collapsed across condition, are very close to what would be expected if selection from the two target categories was random (i.e. 15 runs given 30 target items). Second, it is clear that condition did not influence performance, with the conjunction condition (M = 16.7, SE = 0.3) giving rise to the same number of runs as the feature condition (M = 16.9, SE = 0.29), *t*(11) = 0.38, *p* = 0.71. The same pattern of almost identical feature/conjunction performance was observed with the other dependent variables (see Table 1).

**Table 1:** Summary of behavioural data for Experiments 1 and 2.

		Experiment 1			Experiment 2		
		Runs	ITT	Distance	Runs	ITT	Distance
Feature	**M**	16.9	4597	2.96	16.8	3840	2.78
	**SE**	0.29	188	0.07	0.33	138	0.07
Conjunction	**M**	16.7	4499	2.99	17.8	6671	4.29
	**SE**	0.30	262	0.11	0.44	384	0.10

Table 1 displays the average number of runs, inter-target time (ITT ms) and inter-target distance (ITD meters) as a function of condition.

In Experiment 2, we wanted to increase the search-related task demands, to see if the conjunction condition would influence performance. We did this by changing the target/distractor ratio in the conjunction condition from 50/50 in Experiment 1, to 30/70. We were also concerned that some participants had difficulty using the virtual joystick to navigate. If they were having difficulty moving, this may have biased them to always select the nearest item, regardless of the switch cost. We thus replaced the virtual joystick control with a whole-body movement interface, that pilot testing suggested was more user-friendly.

**Fig 9:**
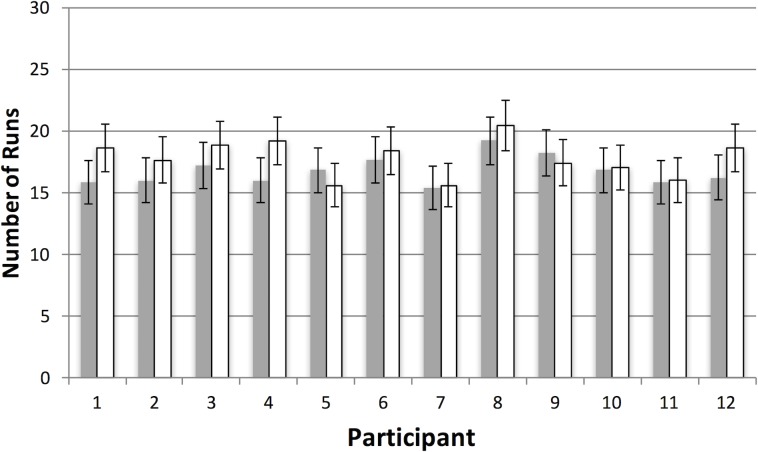
Results for number of runs in Experiment 2. Average number of runs per participant as a function of the feature (grey bars) versus conjunction (white bars) manipulation. Error bars represent one standard error of the mean.

S4 Movie shows the spatial distribution of search episodes in Experiment 2. Fig 9 shows that the target/distractor ratio change had almost no effect on the pattern of run behavior. In fact, participants in the conjunction condition (M = 17.8, SE = 0.44) actually increased their number of runs slightly compared to the feature condition (M = 16.75, SE = 0.33), which still had a 50/50 ratio, *t*(11) = -2.56, *p* < .05. Our prediction, based on previous studies, had suggested that switching would be less common with the added attentional load of conjunction targets.

**Fig 10:**
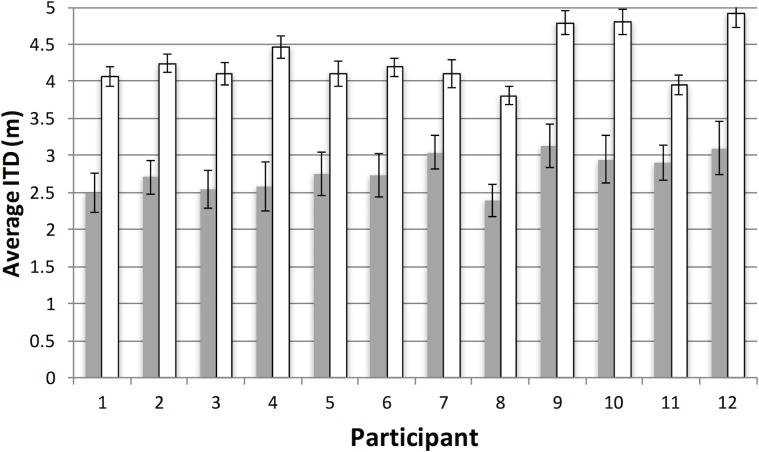
Results for inter-item distance (ITD) in Experiment 2. Average inter-item distance (ITD) per participant as a function of the feature (grey bars) and conjunction (white bars) conditions in Experiment 2. Note that in the conjunction condition, the target/distractor ratio was reduced, so that increased ITDs reflect the sparseness of the displays in these conditions. See text and Table 1 for more details. Error bars represent one standard error of the mean.

**Fig 11:**
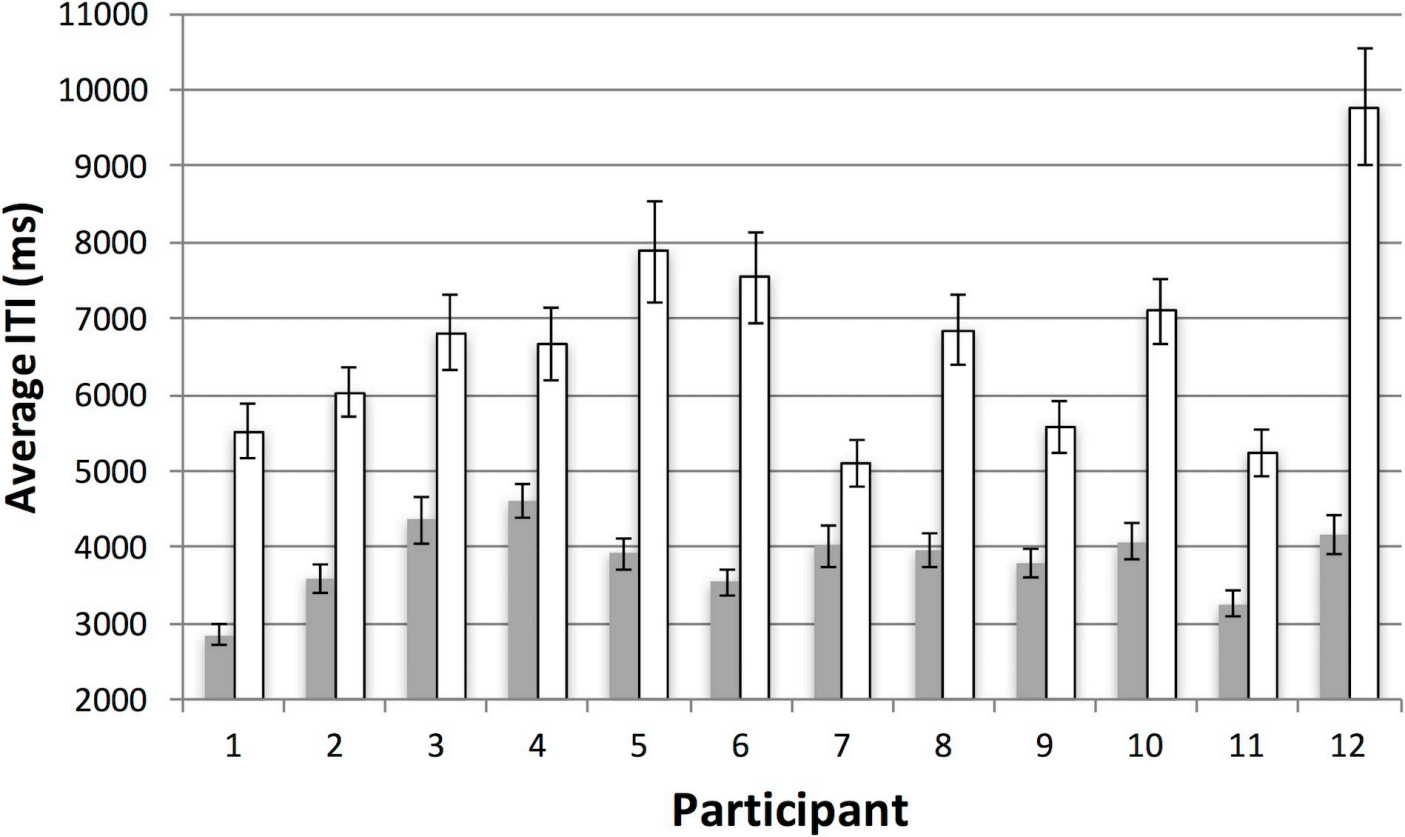
Results for inter-target time (ITT) in Experiment 2. Average inter-target time (ITT) per participant as a function of the feature (grey bars) and conjunction (white bars) conditions in Experiment 2. Note that in the conjunction condition, the target/distractor ratio was reduced, so that increased ITTs reflect the sparseness of the displays in these conditions. See text and Table 1 for more details. Error bars represent one standard error of the mean.

Figs 10 and 11 provide a summary of the spatial and temporal measures in Experiment 2. In Experiment 1, these measures gave rise to very similar performance across the two conditions (Table 1). Here changing the target/distractor ratio appears to have had a clear effect, even though this manipulation did not affect run behavior. Thus, participants moved further in the conjunction condition, (M = 4.29 m, SE = 0.1) than in the feature condition (M = 2.78 m, SE = 0.07), *t*(11) = 19.1, *p* < .001.

They also took longer in the conjunction condition, (M = 6671 ms, SE = 384) than in the feature condition (M = 3840 m, SE = 138), *t*(11) = 8.1, *p* < .001. More interestingly, if we compare the inter-target times (ITTs) in the feature conditions across the two experiments–which both had 50/50 target/distractor ratios–it appears that the use of the motion interface (M = 3840, SE = 138) gave rise to consistently shorter collection episodes than the virtual joysticks (M = 4597, SE = 188), *t*(22) = 3.25, *p* < .01. Again, our methodological change does appear to have been effective, but there was no difference in run patterns.

## Summary & future directions

The goal of this paper was to present our initial findings using a new research tool that provides a search environment modelled on foraging in the wild. Here, we tested the app in two experiments that asked a specific question about human foraging. This question was inspired by the classic laboratory study Marian Dawkins [5] and our previous 2D foraging studies [24], both of which suggested that foraging episodes sometimes consist of extended “runs” where targets are selected from only one category. We wanted to know whether similar run-like behavior would be present in a situation more closely resembling foraging in the wild. Our specific question was whether a conjunction manipulation would modulate behavior.

The results were quite clear. There was no evidence of a shift in foraging behavior between the feature and conjunction conditions in either experiment. Indeed, the number of runs was always very close to what would be expected from random selection between two target categories and there was no evidence of extensive runs. More specifically, in Experiment 1 the feature/conjunction manipulation gave rise to the same number of runs, while in Experiment 2 the number of runs was slightly higher in the conjunction condition. This contradicts our prediction that reducing the number of available targets (from 50% to 30% of available items) would increase the attentional load and reduce the tendency to switch between target categories.

This finding suggests that the extended run behavior found in our original work [24]—which has been replicated a number of times, both by our group [50–52] and others [53–56]—does not rely exclusively on the feature/conjunction manipulation. Rather, it implies that this manipulation interacts with other aspects of the task design and/or display characteristics to constrain foraging behavior. The fact that the influence of the feature/conjunction manipulation appears to be context dependent in this way is in itself very interesting.

Specifically, it raises the possibility that other manipulations commonly used to measure/modulate attention in simplified laboratory tasks might also not generalise to more real-word-like, interactive contexts. However, this does not appear to be the case. For example, Ray Klein and colleagues [18] have recently developed a serious game called the “AttentionTrip” which implements and extends the well-known Attention Network Test [57], as an active, first-person driving scenario. In this dynamic, simulated-3D context they were still able to reliably measure the spatial-compatibility Simon effect [58] as well as measures of attentional orienting, alerting and executive control from the ANT. Similarly, inhibition-of-return (IOR) [59,60], which has long been suggested as a “foraging facilitator” [61] has also be measured in an immersive 3D environment where search episodes took seconds, rather than hundreds of milliseconds [62].

Returning to the current experiments, as already mentioned, they were intended as a first step in examining attention-modulated selection in a complex environment. As such, their design does not allow us to isolate “why” participants continued to switch randomly under conjunction conditions. However, the tool we have developed will make it possible for us–and/or other groups of researchers—to systematically examine the influence of a variety of factors in subsequent studies.

For example, the Unity3D development environment makes it easy to change the viewpoint from first-person to third-person, so a wider, birds-eye, view—more similar to our 2D tasks—could be examined. The complexity of the spatial layout could be systematically varied, such that scene detail could be reduced or even removed completely, leaving only target and distractor items. Rather than active navigation, we could have the player character autonomously drive circuits through the scene, reducing the participants’ active task to choosing between target and distractor items that come within reach. Obviously, the speed of travel, in either a passive or active scenario, could also be varied to change the effective ITT.

Although we can only speculate, we feel that differences in ITT between the 2D and 3D tasks will prove to be a major factor in determining patterns of run behavior. In our 2D tasks, target selection was extremely rapid, leading to an average ITT of approximately 400ms/item. In the 3D game, the need to navigate in the environment in order to find and collect the targets meant that the time interval between successive collection episodes was an order of magnitude higher (around 4 seconds) than in the 2D game. Having made the observation of random conjunction switching using the 3D task, we are, in ongoing experiments, revisiting our 2D tasks, but systematically controlling the “foraging tempo” to further explore the role of temporal constraints on foraging behavior. This approach—taking observations from more naturalistic 3D settings and subsequently testing them in more controlled 2D tasks—illustrates another way in which the tool we are making available here could prove useful for other research groups.

While many of the task manipulations suggested above would require modification to the provided Unity3D project, we should also stress that the working app is flexible enough in several respects to support additional research. In addition to changing experimental parameters (e.g., number and duration of trials, target/distractor ratio), both target and distractor items are fully customizable and the researcher can load any image to appear in the virtual environment. The overall visibility of target and distractor items, their similarity to each other and even their affective or personal significance could all be easily manipulated in this way without the need for any modification to the app.

We should also not lose sight of the fact that the app could prove useful for addressing other more general behavioral questions. Thus, while our experiments focused on run-like foraging behavior, other measures yielded interesting insights into the app and how people use it. For example, the inspection of the distance-travelled and the use of space suggest that participants explore widely in the virtual environment. Memory for the routes taken during exploration, scene-based landmarks, or even the identity of distractor “landmarks”, could all provide useful information for researchers interested in spatial navigation in simulated environments [36].

## Conclusion

To conclude, in the current paper we have introduced a new research tool in the form of a serious game that provides a simulated environment modeled on foraging in the wild. This tool was developed to continue our exploration of attentional constraints on human foraging. Specifically, we were interested in whether a feature/conjunction manipulation could lead to extended run-like behavior in this environment as we previously demonstrated using 2D apps. The answer is clearly that it does not, and we have discussed potential reasons for this difference and avenues for future research aimed at identifying possible causes. However, the app was developed with flexibility in mind and we are optimistic that our group and others will find it a useful tool for further exploring human foraging in a simulated 3D environment.

## Supporting information

S1 File This file contains raw data from each of the 12 participants that took part in Experiment 1. This file is in xlsx format and does contain header information. The column labels (with explanations in parenthesis) are further reported here as follows: 1) PPT (Participant number); 2) Condition (Experimental Condition; feature vs. conjunction); 3) Trial (Trial Number); 4) ClickCount (Number of item collected; 1–30); 5) TargetName (acorn_blue, acorn_red, walnut_red); 6) Z (Target Z Position); 7) Y (Target Y Position); 8) X (Target X Position); 9) ITT (Inter-Target Time); 10) Distance (Inter-Item Distance).
(XLSX)

S2 File This file contains raw data from each of the 12 participants that took part in Experiment 2. This file is in xlsx format and does contain header information. The column labels are the same as for S1 File.
(XLSX)

S1 Movie This movie shows the game in action in the feature condition. (MP4)

S2 Movie This movie shows the game in action in the conjunction condition. (MP4)

S3 Movie This movie shows the spatial distribution of each collection episode for every trial in both conditions of Experiment 1. (MP4)

S4 Movie This movie shows the spatial distribution of each collection episode for every trial in both conditions of Experiment 2. (MP4)
